# Recent Enhancements to Interline and Electron Multiplying CCD Image Sensors †

**DOI:** 10.3390/s17122841

**Published:** 2017-12-07

**Authors:** Eric G. Stevens, Jeffrey A. Clayhold, Hung Doan, Robert P. Fabinski, Jaroslav Hynecek, Stephen L. Kosman, Christopher Parks

**Affiliations:** 1 ON Semiconductor, 1964 Lake Avenue, Rochester, NY 14615, USA; jeffrey.clayhold@onsemi.com (J.A.C.); hung.doan@onsemi.com (H.D.); bob.fabinski@onsemi.com (R.P.F.); stephen.kosman@onsemi.com (S.L.K.); christopher.parks@onsemi.com (C.P.); 2 ON Semiconductor, 2660 Zanker Road, San Jose, CA 95134, USA; jerry.hynecek@onsemi.com

**Keywords:** interline CCD, EMCCD, quantum efficiency, low noise, gain aging

## Abstract

This paper describes recent process modifications made to enhance the performance of interline and electron-multiplying charge-coupled-device (EMCCD) image sensors. By use of MeV ion implantation, quantum efficiency in the NIR region of the spectrum was increased by 2×, and image smear was reduced by 6 dB. By reducing the depth of the shallow photodiode (PD) implants, the photodiode-to-vertical-charge-coupled-device (VCCD) transfer gate voltage required for no-lag operation was reduced by 3 V, and the electronic shutter voltage was reduced by 9 V. The thinner, surface pinning layer also resulted in a reduction of smear by 4 dB in the blue portion of the visible spectrum. For EMCCDs, gain aging was eliminated by providing an oxide-only dielectric under its multiplication phase, while retaining the oxide-nitride-oxide (ONO) gate dielectrics elsewhere in the device.

## 1. Introduction

Although applications of complementary metal oxide semiconductor (CMOS) image sensors have greatly expanded since their introduction, charge-coupled device (CCD) image sensors are still used for their excellent image uniformity (i.e., low fixed pattern noise), and high global-shutter efficiency (GSE).

For capturing quality images in low light, high quantum efficiency and low noise are key attributes for both types of sensors. Achieving high quantum efficiency can be broken up into the following parts:

- Improving micro-lens focusing and fill factor
- Reducing reflection losses
- Reducing absorption losses
- Increasing the collection volume of the photodiodes

To reduce noise, one technique employed on both types of imagers is to improve the charge-to-voltage conversion factor of the output structure. More specific to CCDs, electron multiplication CCD (EMCCD) image sensors were conceived as a way to significantly reduce noise [1]. With an EMCCD, care needed to be taken to ensure that gain was only applied under low-lighting conditions to avoid saturating the device. This put a limit on the intra-scene dynamic range.

**Figure 1:**
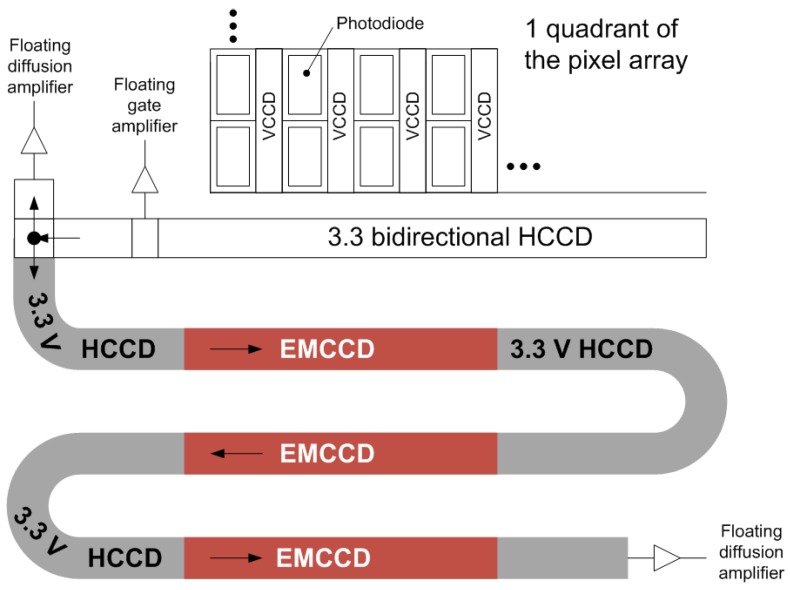
Block diagram of one quadrant of the electron-multiplying charge coupled device (EMCCD) imager as previously reported in Reference [2]. Each EM section contains 300 pixels, with two multiplication phases per pixel for a total of 1800 gain stages.

More recently, an EMCCD image sensor with an output structure that includes a novel, non-destructive floating gate amplifier gain switching architecture for enabling dynamic range improvement has been reported [2]. A block diagram showing one quadrant of the device is shown below in Figure 1. This paper will describe the details of recent process modifications made as reported in Reference [3], that were aimed at improving the sensor’s quantum efficiency and smear via improved photodiode (PD) collection volume, elimination of the gain-aging artifact [2,3,4,5], reduced PD-to-VCCD transfer gate voltage, reduced electronic shutter voltage, and reduced point defects.

## 2. Improved Quantum Efficiency

**Figure 2:**
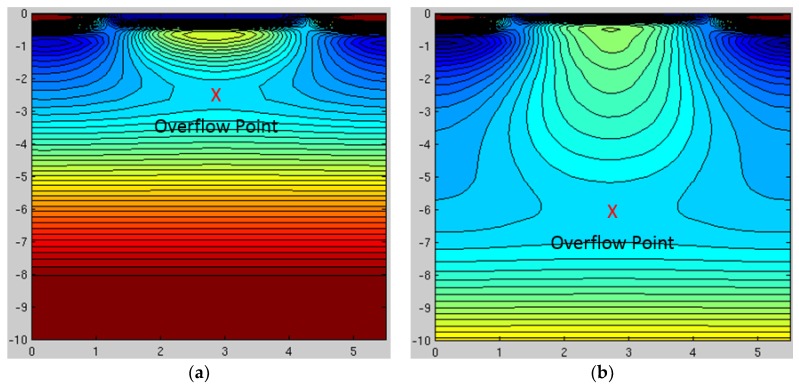
Comparison of potential contours for the old and new processes. Images show two-dimensional (2-D) cuts from the three-dimensional (3-D) model space of image area pixel shown in Figure 1, which has half a vertical-charge-coupled-device (VCCD) on the left, a photodiode (PD) in the middle, and another half VCCD on the right. (**a**) Old process. (**b**) New process.

**Figure 3:**
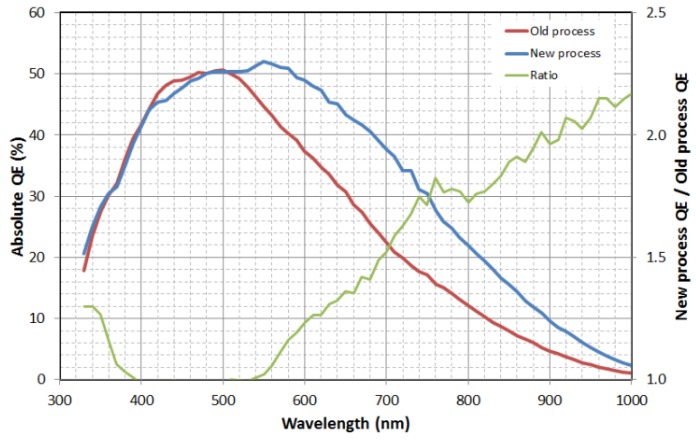
Measured absolute quantum efficiency for the old and new processes.

Quantum efficiency (QE) is improved by a factor of over 2× in the NIR region of the spectrum by increasing the photodiode’s collection volume via the use of high-energy MeV implantation for both the deep p-well (up to 6.5 MeV) and deep n-type photodiode regions (up to 10 MeV). Comparison of the electrostatic potential contours of the old and new processes are shown below in Figure 2. As can be seen, the overflow depth is over 6 µm with the new MeV implant process vs. only around 2.5 µm deep for the old process. The resulting improvement in absolute quantum efficiency is shown below in Figure 3, which is in good agreement with a simple Beer-Lambert absorption law [6].

The data of Figure 3 show that the quantum efficiency improvement is concentrated at longer wavelengths as would be expected from the Beer-Lambert law. Because the absorption coefficient of silicon at short wavelengths is quite high, no improvement is anticipated in the blue from the deep MeV implants. The small QE differences around 420–480 nm are merely due to part-to-part variation.

## 3. Masking of High-Energy Implants

### 3.1. Patterning

**Figure 4:**
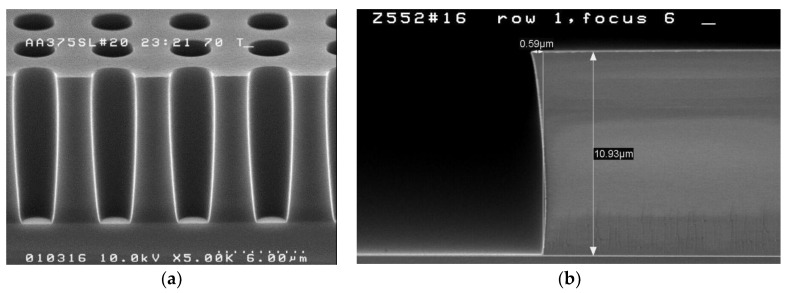
Cross-sections of photoresist patterns. (**a**) Cross-section of the photodiode extension (PDext) resist pattern within the pixel array. (**b**) Cross-section of the deep p-well (PWELL) resist at the array edge.

**Figure 5:**
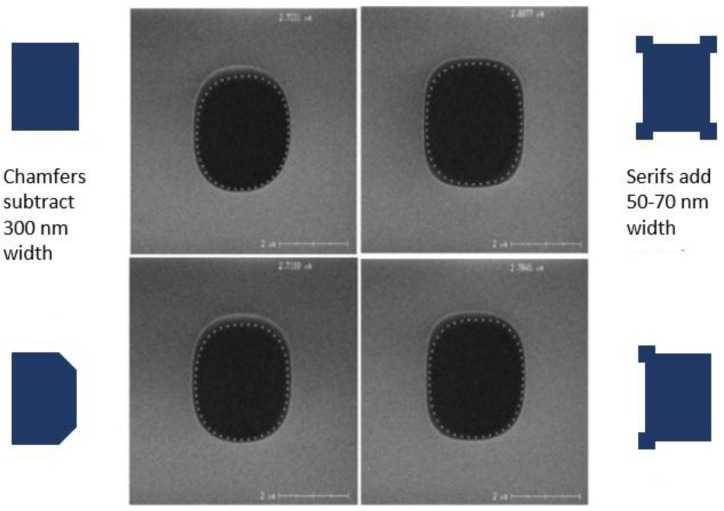
Effects of chamfers and serifs on the dimensions of the photoresist used to mask the photodiode extension (PDext) implant. The openings are approximately 2.5 μm by 3.3 μm.

One of the challenges of using high-energy implantation is masking. A chemically-amplified thick photoresist is used for the high aspect ratio masking of both MeV implants. The photoresist was exposed using a wide-field, low N.A., i-line stepper, and developed with a metal-ion-free developer. Alignment measurement was improved by thinning the resist over the measurement structures with a second low-dose exposure. Figure 4a shows a 4.25:1 aspect ratio thick resist image for the masking of the deep MeV n-type photodiode extension (PDext) implants within the pixel array of Figure 1. Figure 4b is an SEM of the sidewall of the thick photoresist at the edge of the deep MeV p-well (PWELL) pattern, created using the same lithographic processes. Stepper focus was optimized to minimize the reentrance of the top and foot of the photoresist sidewall. The PDext design was optimized by the addition of serifs. Figure 5 is a comparison of top-down SEM images, showing the impact of serifs and chamfers on the shape of the PDext opening in the resist. Dimensional control of the opening is important because it affects several key performance attributes of the photodiode. 

### 3.2. Radiation

One of the concerns presented by high-energy implants in the MeV range is potential radiation activity. Target materials, including the photoresist, can become activated. Carbon is a primary constituent of many photoresists. During phosphorus implantation, the coulomb barrier for ^31^P-^12^C nuclear activation is nearly 40 MeV, much higher than the capability of the implanter. So, there is no radiation safety risk from this interaction. However, for boron implantation, the ^11^B-^12^C coulomb barrier is only about 10 MeV, which is easily attainable and therefore presents a radiation concern.

A careful examination of the radiation activity for ^11^B-^12^C as characterized by different institutions was compiled by Nick White in Reference [7]. As the energy approaches the coulomb barrier, radiation activity increases by an order of magnitude per MeV. The recommended safe exposure rate limit for a human operator is about 0.6 μS/h. This limits the boron energy to just over 7 MeV using a carbon based photoresist mask. Accordingly, we capped our boron implant energy at 6.5 MeV. Radiation activity is not expected to be a problem. Refer to Table 1. If a higher atomic number were to be used for the mask material, a higher boron implant energy would be possible. Because the implanter beam stop and aperture typically use graphite, the same ^11^B-^12^C radiation activity concern applies, independent of the choice of photoresist. 

## 4. Improved Reliability

**Figure 6:**
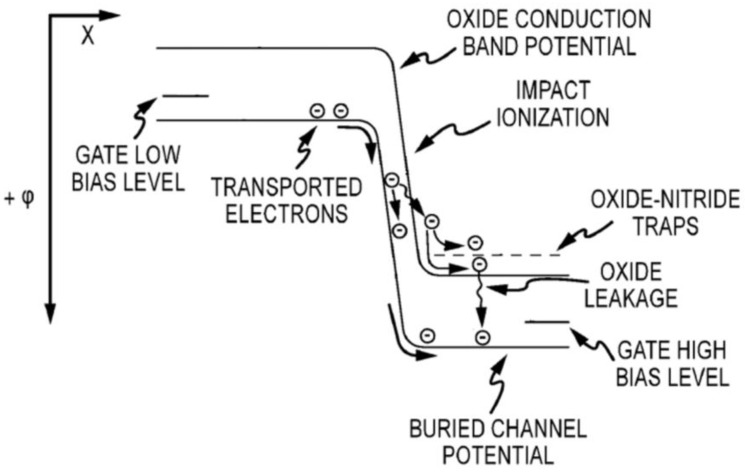
Conduction band diagram along the EMCCD illustrating the physical mechanism of gain degradation. Note that the conduction band of the buried channel in the silicon is labeled as “BURIED CHANNEL POTENTIAL”.

Stability of the multiplication gain in EMCCDs is a known problem, as has been previously reported [2,3,4,5]. This loss of gain results from the buildup of hot electrons being injected into the gate dielectric over time during normal device operation, as illustrated in the conduction band diagram of Figure 6 below. As an additional complication, the more signal electrons that are present, the more can be injected into the dielectric, resulting in this drift in gain being a function of signal level. 

**Figure 7:**
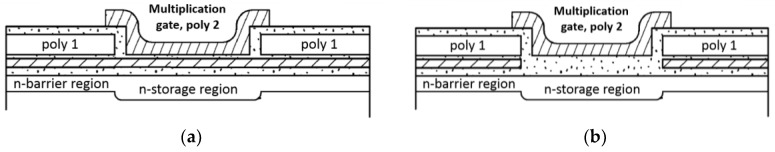
Comparison of dielectric structures under the multiplication gates: (**a**) old structure with oxide-nitride-oxide (ONO); (**b**) new structure with oxide-only under the high-field gates.

**Figure 8:**
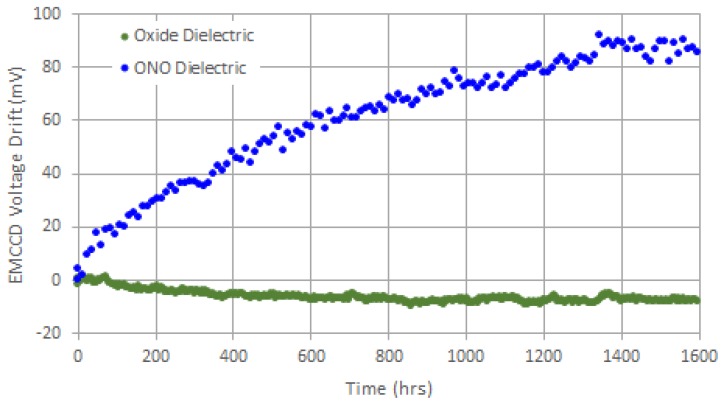
Shift in multiplying gate voltage required for 20× gain versus time for old and new processes. The slight drift downward in the oxide-only dielectric curve was due to some slight inaccuracy in the temperature controller.

To get around this problem, we incorporated an oxide-only dielectric structure under the high-field, multiplication gates of the EMCCD registers, as shown above in Figure 7b. The original gate structure is shown for comparison in Figure 7a. As can be seen below in Figure 8, gain aging was eliminated for the new process using an oxide-only dielectric under the high-field, multiplication gates.

## 5. Improved Transfer Efficiency

**Figure 9:**
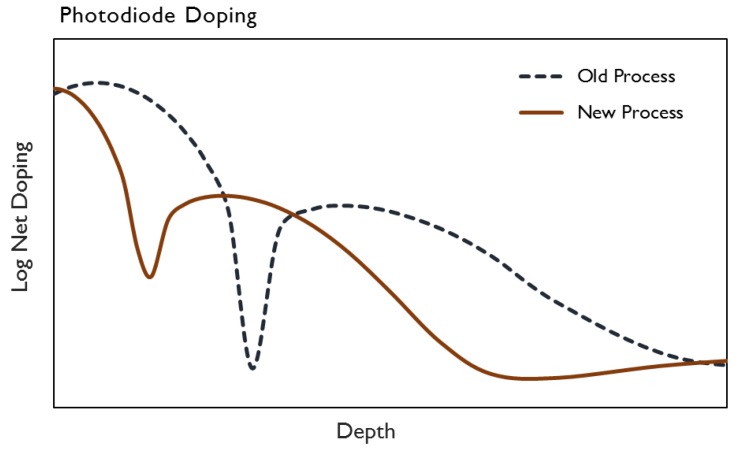
Comparison of doping profiles for old and new shallow photodiode implants.

By reducing the thickness of the photodiode’s p+ pinning layer along with the depth of its shallow, higher dose n-type component, a given charge capacity can be achieved with a lower empty photodiode potential and nominal substrate bias. The thin, spike pinning layer was formed via low-energy BF_2_ implantation, whereby only the steep concentration gradient tail of the distribution is within the silicon. The shallow, higher dose n-type photodiode implant is formed via As implantation. A relative comparison of the doping profiles between the new and old processes are shown below in Figure 9.

**Figure 10:**
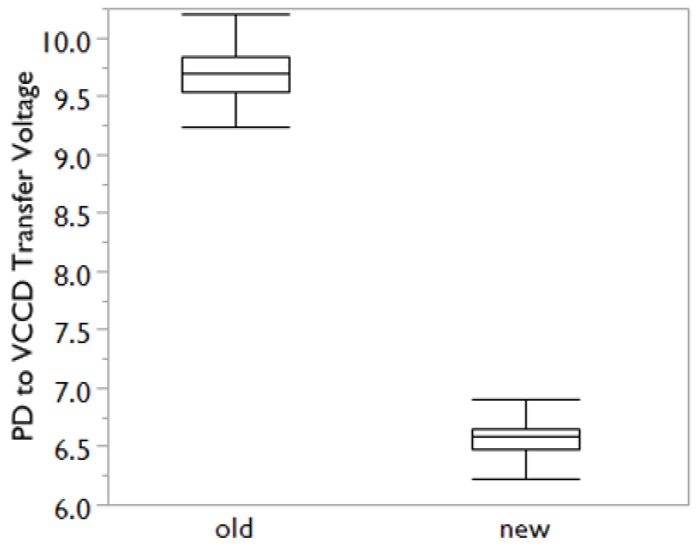
Comparison of the minimum voltage necessary to transfer charge from the photodiode to the vertical shift register (VCCD). The new process shows significant improvement, lowering the voltage by 3 V.

The shallower surface layer construction of the photodiode is also found to reduce the parasitic wells and barriers at the transfer gate edge (examples of such wells and barriers can be found Reference [8]). By reducing these transfer barriers and the empty diode potential, the transfer-gate voltage required for complete charge transfer from the PD to the VCCD was reduced by 3 V, as shown below in Figure 10. With the combination of a lower empty diode potential and nominal substrate bias, the voltage on the n-type substrate required to clear the photodiode during electronic shutter operation was reduced by 9 V.

## 6. Reduced Point Defects

**Figure 11:**
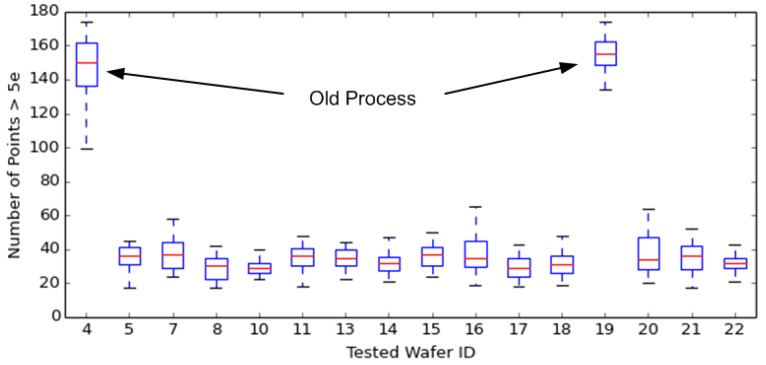
Point defects >5 e^−^ at 0 °C for 2 Mp imagers with old and new shallow photodiode (PD) implants.

Quite independent of the deep MeV photodiode implants, changes were made to the lower energy photodiode implants (as shown above in Figure 9) that also improved image quality. It was found that modifying the shallow photodiode implants, using BF_2_ to form the p+ pinning layer and As for the high-dose shallow photodiode implant, reduced point defects by about 5×, as shown below in Figure 11.

## 7. Reduced Image Smear

**Figure 12:**
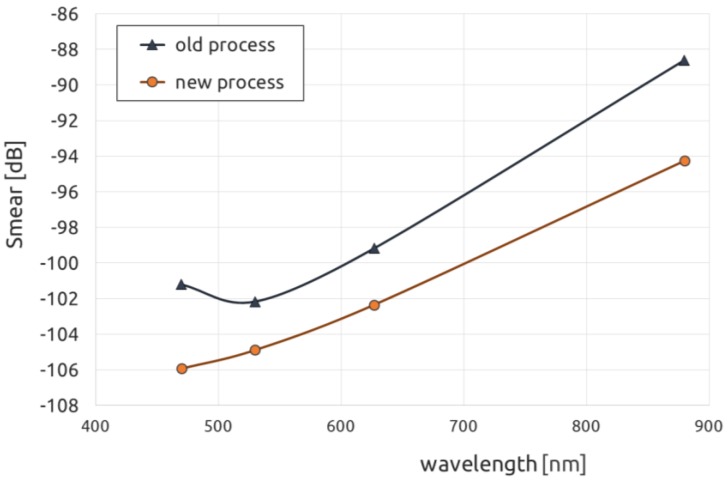
Improved smear performance with photodiode extension implants and shallower BF_2_ pinning layer.

Image quality was also improved by reducing image smear [9], as shown below in Figure 12. This was accomplished in two ways. At longer wavelengths, smear is reduced via the improved collection efficiency of the photodiode. Because smear is essentially given as a ratio of the undesired signal that leaks into the VCCD while it is being read out to that of the photosignal collected in the photodiode, a 2× increase in QE will be followed by a commensurate drop of 6 dB in smear, for example. At shorter wavelengths, reducing the thickness of the photodiode’s p+ pinning layer reduces smear. This is because as the quasi-neutral region in the pinning layer is made thinner, there will be fewer photoelectrons generated there that can subsequently diffuse into the VCCD [10]. From earlier work on other devices without the MeV implants, we anticipated an improvement of blue smear between 2 and 5 dB, without any measureable difference in green, red, or NIR smear from the thinner pinning and shallow n-type photodiode implants. It should be noted that, because the smear signal is extremely small, no measurable difference in blue sensitivity was found, as expected.

## 8. Conclusions

**Figure A1:**
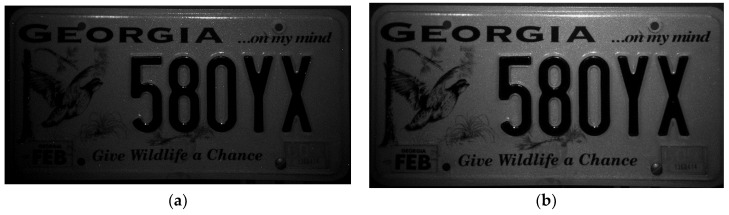
Sample images with NIR illuminator and no EMCCD gain. (**a**) Old process. (**b**) New process.

**Figure A2:**
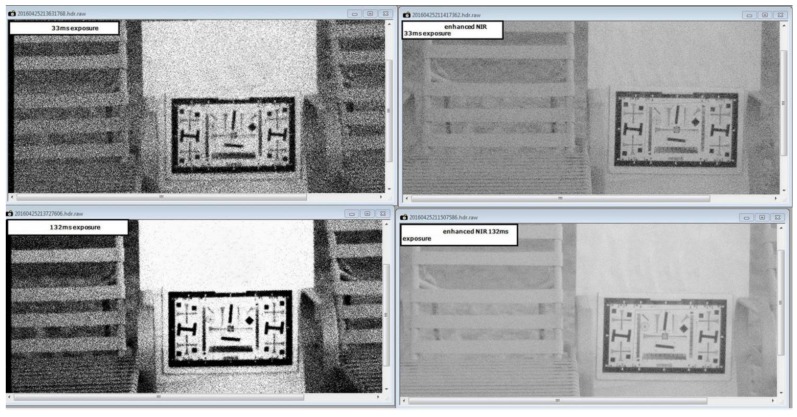
Sample images taken just after dusk on beach in Delray, FL, USA. Captured with Imperx Puma P1940 camera. Images from a device with the old process is shown on the left, the enhanced NIR process is on the right.

Recent process changes to improve the performance of our EMCCD devices have been successfully demonstrated. These improvements resulted in increased quantum efficiency, elimination of gain aging, and reduced PD-to-CCD transfer and electronic-shutter voltages. A performance summary is given below in Table 2, and some sample images are shown in Figure A1 and Figure A2 in Appendix A.

## Figures and Tables

**Table 1:** Estimated radiation activity after implanting 6.5 MeV ^11^B into photoresist.

Boron Dose/cm^−2^	Bq/cm^2^	Bq for 150 mm Wafer
1.0 × 10^11^	1.0 × 10^−7^	1.0 × 10^−5^
1.0 × 10^12^	1.0 × 10^−6^	1.0 × 10^−4^
1.0 × 10^13^	1.0 × 10^−5^	1.0 × 10^−3^

**Table 2:** Device performance summary table.

Parameter	Old Process	New Process
pixel size (µm)	5.5	5.5
charge-to-voltage (µV/e^−^)	44	44
charge capacity, nominal (ke^−^)	20	20
quantum efficiency at 450, 550, 650, 900 nm (%)	49, 45, 31, 5	48, 52, 43, 10
absolute electronic shutter voltage (V)	27	18

## Appendix A

Additional images demonstrating improved sensor performance are shown in this appendix.

## References

[1] Hynecek J. (1992). CCM—A New Low-Noise Charge Carrier Multiplier Suitable for Detection of Charge in Small Pixel CCD Image Sensors. IEEE Trans. Electron Devices.

[2] Parks C., Kosman S., Nelson E., Roberts N., Yaniga S. A 30 fps 1920 × 1080 Pixel Electron Multiplying CCD Image Sensor with Per-Pixel Switchable Gain. Proceedings of the 2015 International Image Sensor Workshop (IISW).

[3] Stevens E.G., Clayhold J.A., Doan H., Fabinski R.P., Hynecek J., Kosman S.L., Parks C. Recent Enhancements to Electron Multiplying CCD Image Sensors. Proceedings of the 2017 International Image Sensor Workshop (IISW).

[4] De Monte B., Bell R.T. Development of an EMCCD for LIDAR applications. Proceedings of the International Conference on Space Optics, Session 28.

[5] Ingley R., Smith D.R., Holland A.D. (2009). Life Testing of EMCCD Gain Characteristics. Nucl. Instrum. Methods Phys. Res. Sect. A.

[6] Jenkins F., White H.E. (2001). Fundamentals of Optics.

[7] White N.R., Tokoro N., Bell E. (2008). Radiation Issues Surrounding Very High Energy Ion Implantation. AIP Conference Proceedings.

[8] Burkey B.C., Chang W.C., Littlehale J., Lee T.H., Tredwell T.J., Lavine J.P., Trabka E.A. The Pinned Photodiode for an Interline-Transfer CCD Image Sensor. Proceedings of the 1984 IEEE International Electron Devices Meeting.

[9] Kuroda T., Kuriyama T., Matsuda Y., Kozono T., Matsumoto S., Hiroshima Y., Horii K. A Smear-Suppressing CCD Imager. Proceedings of the 1986 IEEE International Solid-State Circuits Conference, Digest of Technical Papers.

[10] Tanabe A., Kudoh Y., Kawakami Y., Masubuchi K., Kawai S., Yamada T., Morimoto M., Arai K., Hatano K., Furumiya M. (2000). Dynamic Range Improvement by Narrow-Channel Effect Suppression and Smear Reduction Technologies in Small Pixel IT-CCD Image Sensors. IEEE Trans. Electron Devices.

